# Right ventricular free wall longitudinal strain is independently associated with mortality in mechanically ventilated patients with COVID-19

**DOI:** 10.1186/s13613-022-01077-7

**Published:** 2022-11-12

**Authors:** James McErlane, Philip McCall, Jennifer Willder, Colin Berry, Ben Shelley, the COVID-RV investigators, A. Reece, C. Kitchen, M. Gillies, V. Dabek, V. Irvine, J. MacBrayne, K. Sim, T. Scott, E. Trumper, F. Savage, A. Allan, J. Falconer, A. Coutts, A. McDonald, J. Rutherford, D. Christie, C. Jardine, A. Puxty, M. Hughes, S. Cathcart, M. Sim, B. Docking, M. Thornton, B. Greatorex, J. Rae, C. Barr, C. Bradley, F. Barrett, R. Campbell, N. Clarke, M. Mascarenhas, J. Matheson, D. McDonald, M. O Hara, L. O keeffe, L. Gemmell, R. Price, M. McHendry, D. McLaughlan, C. Herman, H. Elliot, S. Meehan, J. Allan, D. Finn, G. Brannan, S. Wood, T. Watson, K. Ross, N. Tatarkowska, R. Boyle, E. Lee, D. Strachan, A. Morrison, P. Lucie, C. Lochrin, S. Clements, D. Vigni, B. Stanley, C. M. Messow

**Affiliations:** 1 Anaesthesia, Critical Care & Peri-Operative Medicine Research Group, University of Glasgow, Glasgow, UK; 2 Department of Anaesthesia, Golden Jubilee National Hospital, Clydebank, UK; 3 West of Scotland School of Anaesthesia, NHS Education for Scotland, Glasgow, UK; 4 Institute of Cardiovascular and Medical Sciences, University of Glasgow, Glasgow, UK

**Keywords:** Right ventricle, Speckle tracking echocardiography, Coronavirus disease 2019, Mechanical ventilation

## Abstract

**Background:**

Right ventricular (RV) dysfunction has been commonly reported in patients with Coronavirus disease 2019 (COVID-19), and is associated with mortality in mixed cohorts of patients requiring and not requiring invasive mechanical ventilation (IMV). Using RV-speckle tracking echocardiography (STE) strain analysis, we aimed to identify the prevalence of RV dysfunction (diagnosed by abnormal RV-STE) in patients with COVID-19 that are exclusively undergoing IMV, and assess association between RV dysfunction and 30 day mortality. We performed a prospective multicentre study across 10 ICUs in Scotland from 2/9/20 to 22/3/21. One-hundred-and-four echocardiography scans were obtained from adult patients at a single timepoint between 48 h after intubation, and day 14 of intensive care unit admission. We analysed RV-STE using RV free-wall longitudinal strain (RVFWLS), with an abnormal cutoff of  > −20%. We performed survival analysis using Kaplan–Meier, log rank, and multivariate cox-regression (prespecified covariates were age, gender, ethnicity, severity of illness, and time since intubation).

**Results:**

Ninety-four/one-hundred-and-four (90.4%) scans had images adequate for RVFWLS. Mean RVFWLS was −23.0% (5.2), 27/94 (28.7%) of patients had abnormal RVFWLS. Univariate analysis with Kaplan–Meier plot and log-rank demonstrated that patients with abnormal RVFWLS have a significant association with 30-day mortality (*p* = 0.047). Multivariate cox-regression demonstrated that abnormal RVFWLS is independently associated with 30-day mortality (Hazard-Ratio 2.22 [1.14–4.39], *p* = 0.020).

**Conclusions:**

Abnormal RVFWLS (> −20%) is independently associated with 30-day mortality in patients with COVID-19 undergoing IMV. Strategies to prevent RV dysfunction, and treatment when identified by RVFWLS, may be of therapeutic benefit to these patients.

*Trial Registration*: Retrospectively registered 21st Feb 2021. ClinicalTrials.gov Identifier: NCT04764032.

**Supplementary Information:**

The online version contains supplementary material available at 10.1186/s13613-022-01077-7.

## Background

Coronavirus disease 2019 (COVID-19) has caused a worldwide pandemic since March 2020. In its most severe form, COVID-19 can present as acute respiratory distress syndrome (ARDS), requiring invasive mechanical ventilation (IMV). Right ventricular (RV) dysfunction is a common finding in patients with severe acute respiratory failure both in those requiring, and not requiring IMV [1–7]. Two-dimensional RV speckle-tracking echocardiography (STE) longitudinal strain analysis is a novel parameter, recently highlighted by expert opinion as an important but underutilised measure of RV function in patients on the intensive care unit (ICU) [8]. RV-STE has been shown to be independently associated with mortality in COVID-19 patients, where other conventional RV echocardiography parameters have not [4, 9]. STE studies investigating RV dysfunction (RVD) in patients with COVID-19 have, however, been limited by small sample size, retrospective design, the use of clinically necessitated echocardiography scans (e.g., for cardiovascular instability), and varying requirement for IMV [10, 11]. It is unclear how severe COVID-19 requiring ICU admission and the potentially deleterious effects of IMV, impact upon RV function and outcomes in patients with COVID-19. To address this question, and the limitations of previous studies, we designed a prospective, ICU clinician delivered, multicentre, echocardiography study; Right Ventricular Dysfunction in Ventilated Patients with COVID-19 (COVID-RV) [12]. The primary analysis of COVID-RV demonstrated a prevalence of RVD (defined as severe RV dilation and interventricular septal flattening, i.e., severe acute cor pulmonale) of 6% and an association between RVD and 30-day mortality (*p* = 0.05). To determine the utility of RV-STE in this cohort, we performed an a-priori defined secondary analysis of COVID-RV using RV-STE, seeking association between RVD and outcomes, and further examining potential causative mechanisms of RVD in patients with COVID-19 [13].

## Methods

### Study setting and population

Study protocol and methods have been previously published [12, 13]. We performed a prospective observational multicentre cohort study across ten ICUs in NHS Scotland. Ethics approval was obtained from Scotland A Research Ethics Committee (with approval for consent under the Adults with Incapacity Act, 2000-20/SS/0059). We obtained informed consent from a legal representative for all patients. Inclusion criteria included patients with a confirmed diagnosis of COVID-19 who were more than 16 years old requiring IMV for severe acute respiratory failure. Exclusion criteria included: pregnancy, extracorporeal membrane oxygenation (ECMO) for respiratory or cardiovascular failure, prior participation in COVID-RV, ongoing participation in research that may undermine the scientific basis of the study, and end of life care (where the patient was not expected to survive longer than 24 h). COVID-RV was registered at ClinicalTrials.gov (NCT04764032).

### Data

Study data were collected and stored electronically on REDCap (Vanderbilt University, Nashville, United States of America), hosted by the University of Glasgow.

#### Clinical and laboratory data

All data were collected prospectively. This included: baseline demographics, chronic comorbidities, acute comorbidities since hospital admission, severity of COVID-19 illness, clinical data relating to potential causative mechanisms for RVD, and follow-up data. On the day of echocardiography, patients had blood samples taken for high sensitivity troponin (hsTn) (T or I, subject to the assay used at each site) and N-terminal pro B-type natriuretic peptide (NT-proBNP). Abnormal values were defined for troponin (hsTnT ≥ 15 ng/L or hsTnI ≥ 34 ng/L for males; ≥ 16 ng/L for females) and for NT-proBNP (> 300 ng/L) [14–16].

#### Echocardiography

A single transthoracic echocardiography (TTE) scan was performed between 48 h after intubation, and day 14 of ICU admission for each patient. This was performed as soon as was feasible after recruitment. To reflect clinical practice in ICU, imaging was in keeping with the protocol for a focused intensive care echocardiography (FICE) scan [17]. FICE scanning uses 2D TTE images to rapidly assess for significant cardiac pathology in intensive care patients, and includes a parasternal short axis, parasternal long axis, apical four-chamber (A4C), and a subcostal view. In addition, we requested an RV focussed A4C view for RV-STE analysis; with four beats, electrocardiography monitoring, and a frame rate of 60–80 frames/second. Offline RV-STE strain, and conventional RV echocardiography analysis, was performed using vendor neutral TomTec (Unterschleißheim, Germany) 2D-Cardiac Performance Analysis (2D-CPA). Images were analysed in a randomised order by reporters blinded to clinical data. RV-STE analysis was conducted in accordance with recent consensus guidelines [18]. A single beat was used for RV-STE analysis, if atrial fibrillation was present, an average of three beats was used. Semi-automated speckle tracking was performed, with manual adjustments to the endocardial contours to ensure adequate tracking of the endocardium. Peak RV free-wall (RVFWLS—average of the free-wall apical, middle, and basal segments), and peak RV four-chamber longitudinal strain (RV4CSL—average of the combined six segments from free-wall and septum) were reported. RV fractional area change (FAC) was reported in conjunction with the 2D-CPA strain analysis. RVD was defined by the abnormal cutoff for RVFWLS of  > −20% in accordance with American Society of Echocardiography guidelines [19] and previous studies [5, 20–22]. Scans were excluded if they did not include an A4C view, and where image quality did not allow all RV segments to be adequately tracked.

Where the collected image set allowed, offline analysis was undertaken for tricuspid annular plane systolic excursion (TAPSE), S’ wave velocity at the tricuspid annulus (S’), right ventricular index of myocardial performance (RIMP), and left ventricular (LV) eccentricity index (LVEI) in accordance with current British Society of Echocardiography (BSE) guidelines [23]. Echocardiographer specialty (intensive care clinician, cardiologist, or cardiac physiologist/departmental echocardiographer) and accreditation (none, FICE, FICE mentor, BSE Critical Care accreditation, BSE full accreditation, or other) was recorded.

#### Feasibility and reproducibility

Feasibility of RVFWLS was defined as the percentage of scans with images of sufficient quality for RVFWLS analysis. Twenty randomly selected scans were re-reported by the same reporter 2 weeks after initial reporting and were reported by a second reporter to allow assessment of intra- and inter-observer agreement. Reproducibility was assessed by intraclass correlation co-efficient (ICC) using two-way mixed effects with absolute agreement, and Bland–Altmann plots (mean bias and limits of agreement [LOA]).

#### Outcomes

The primary outcome was association between RVD and 30-day mortality from ICU admission. Secondary outcomes included; need for renal replacement therapy (RRT), need for prone ventilation, and requirement for ECMO referral at 30 days. Exploratory outcomes investigated for association between possible causative mechanisms and abnormal RVFWLS. Previously reported power simulations, assuming a prevalence of RVD of 25–50% and mortality of 50%, suggested  ≥ 80% power in most scenarios [13].

#### Statistical considerations

Continuous data are presented as mean (standard deviation [SD]) or median (interquartile range [IQR]). Ordinal and categorical data are presented as *n* (%). Between group differences were analysed using Student's *T* test or Mann–Whitney test for continuous variables, categorical variables were analysed using Chi Squared-test or Fisher’s Exact test. Univariate survival analysis was performed using Kaplan–Meier plot and log-rank analysis. Multivariate cox regression sought an independent association between abnormal RVFWLS and 30-day mortality with an a-priori analysis plan to adjust for patient demographics (age, gender, ethnicity), phase of disease (time from intubation to echocardiography) and baseline severity of illness (Acute Physiology And Chronic Health Evaluation II score within 24 h of ICU admission) [13]. Variables in the cox regression were assessed for an interaction between time variable and covariate to establish that the proportional hazard’s function assumption was met. Statistical analyses were performed using SPSS version 28.0.0.0 (IBM, United States of America). A two-sided *p* < 0.05 was considered statistically significant.

## Results

One-hundred-and-twenty-one patients were recruited to COVID-RV between 2/9/2020 and 22/3/2021. Three patients were excluded after recruitment—two due to technical factors preventing echocardiography, one was extubated prior to echocardiography. Due to technical issues in storage and transfer, we were unable to obtain echocardiography scans for offline analyses for 14 patients, resulting in 104 scans for RV-STE analysis (Additional file 1: Figure S1). The median age of patients who had RV-STE reported was 59 years [53, 67.3], and 57 (60.6%) patients were male (Table 1).

**Table 1:** Patient characteristics from hospital admission to day of Echocardiography

		All (*n* = 94)	Normal RVFWLS (≤ -20%) (*n* = 67)	Abnormal RVFWLS (> −20%) (*n* = 27)	*p* value
Age, years		59 [53, 67.3]	60 [54, 68]	58 [50, 66]	0.362^§^
Male		57 (60.6%)	41 (61.2%)	16 (59.3%)	0.862^*^
BMI, kg/m^2^	n (n missing)	92 (2) 31.6 [29.5, 36.2]	66 (1) 32.2 [30.0, 36.8]	26 (1) 30.8 [28.0, 34.2]	0.206^§^
Ethnicity					
White		82 (87.2%)	60 (89.6%)	22 (81.5%)	0.231^ω^
Non-white		12 (12.8%)	7 (10.4%)	5 (18.5%)	
Clinical frailty score	n (n missing)	93(1) 2 [2, 3]	66 (1) 2 [2, 3]	27 (0) 2 [2, 3]	0.369^§^
APACHE II score	n (n missing)	89 (5) 16 [13, 19]	63 (4) 16 [14, 19]	26 (1) 14.5 [11, 18.25]	0.103^§^
CCCC	n (n missing)	87 (7) 10.3 (2.8)	63 (4) 10.5 (2.6)	24 (3) 9.7 (3.1)	0.189^η^
*Comorbidities*					
Smoking					
Non-smoker		53 (56.4%)	39 (58.2%)	14 (51.9%)	0.837^*^
Ex-smoker > 1 year		34 (36.2%)	23 (34.3%)	11 (40.7%)	
Current or within 1 year		7 (7.4%)	5 (7.5%)	2 (7.4%)	
Alcohol history					
n (n missing)		92 (2)	65 (2)	27 (0)	0.945^*^
None		33 (35.9%)	24 (36.9%)	9 (33.3%)	
Minimal		45 (48.9%)	32 (49.2%)	13 (48.1%)	
Moderate		6 (6.5%)	4 (6.2%)	2 (7.4%)	
Excess		8 (8.7%)	5 (7.7%)	3 (11.1%)	
Hypertension		30 (31.9%)	22 (32.8%)	8 (29.6%)	0.763^*^
Coronary artery disease		8 (8.5%)	5 (7.5%)	3 (11.1%)	0.235^*^
Diabetes		31 (33%)	20 (29.9%)	11 (40.7%)	0.310^*^
Asthma		12 (12.8%)	7 (10.4%)	5 (18.5%)	0.316^ω^
COPD		7 (7.4%)	5 (7.5%)	2 (7.4%)	0.815^*^
*Treatments before intubation*					
Intravenous corticosteroids		62 (66%)	43 (64.2%)	19 (70.4%)	0.567^*^
Non-invasive ventilation		65 (69.1%)	46 (68.7%)	19 (70.4%)	0.871^*^
High flow nasal oxygen		50 (53.2%)	35 (52.2%)	15 (55.6%)	0.771^*^
Awake self-proning		46 (48.9%)	31 (46.3%)	15 (55.6%)	0.415^*^
*Acute comorbidities*					
New arrhythmias		16 (17.0%)	11 (16.4%)	5 (18.5%)	0.271^*^
Confirmed or suspected PTE	Radiologically confirmed Clinically suspected No Unknown	4 (4.3%) 4 (4.3%) 84 (89.4%) 2 (2.1%)	1 (1.5%) 4 (6.0%) 61 (91.0%) 1 (1.5%)	3 (11.1%) 0 (0.0%) 23 (85.2%) 1 (3.7%)	0.097^*^
Acute coronary syndrome		5 (5.3%)	3 (4.5%)	2 (7.4%)	0.623^ω^
Requirement for RRT		14 (14.9%)	8 (11.9%)	6 (22.2%)	0.117^*^
Requirement for prone invasive ventilation		61 (64.9%)	43 (64.2%)	18 (66.7%)	0.745^*^

Data are presented as mean (SD), median [IQR] or n (%). Data are complete unless indicated by n (n missing)

*RVFWLS* Right Ventricular free-wall longitudinal strain, *BMI* Body Mass Index, *APACHE* Acute Physiology And Chronic Health Evaluation, *CCCC* Coronavirus Clinical Characterisation Consortium, *COPD* Chronic Obstructive Pulmonary Disease, *PTE* Pulmonary Thromboembolism, *ACS* Acute Coronary Syndrome, *RRT* Renal Replacement Therapy

Between-group differences were assessed using Student’s *T* test (η), Mann–Whitney *U* test (§), Fisher’s Exact test (ω), and Pearson Chi-Square test (*)

### Feasibility and reproducibility

Ninety-four out of 104 scans had images of sufficient quality for RVFWLS analysis, giving an overall feasibility of 90.4%. ICU clinicians performed 76.9% (80/104) of scans. There was no difference in feasibility between images acquired by echocardiographers of different levels of accreditation (p = 0.672, Additional file 1: Table S1). RVFWLS showed excellent intra-observer reproducibility: ICC 0.91 (*p* < 0.001). Bland–Altman analysis demonstrated a mean bias of −1.24% (LOA 5.39%, −6.87%). RVFWLS also had very good inter-observer reproducibility: ICC 0.88 (*p* < 0.001) with a mean bias of 0.52% (LOA 7.40%, −8.44%).

### RVFWLS analysis and other echocardiography parameters

Mean RVFWLS was −23.0% (5.2%). Twenty-seven patients (28.7%) had abnormal RVFWLS (> −20%). Patients with abnormal RVFWLS had a median of 21 days [16, 27.5] from symptom onset to echocardiography, significantly longer than patients with normal RVFWLS (18 days [13, 21], *p* = 0.011). There was no difference in time from intubation to echocardiography, with a median of 5 days [3, 9] in abnormal RVFWLS and 5 days [4, 8] in normal RVFWLS groups (*p* = 0.794 Table 2, see Additional file 1: Figure S2 for distribution of time from intubation to echocardiography). Patients with abnormal RVFWLS had significantly lower RV4CSL, RVFAC, TAPSE, S’, and higher RIMP values (*p* ≤ 0.002 for all, Table 2). There was no difference in the prevalence of subjective LV dysfunction between normal and abnormal RVFWLS groups (*p* > 0.999, Table 2).

**Table 2:** Echocardiography parameters

Echocardiography parameter		All (*n* = 94)	Normal RVFWLS (≤−20%) (*n* = 67)	Abnormal RVFWLS (> −20%) (*n* = 27)	*p* value
Time from symptom onset to echocardiography (days)	n (n missing)	93 (1) 18 [13.5, 22]	67 (0) 18 [13, 21]	26 (1) 21 [16, 27.5]	0.011^§^
Time from intubation to echocardiography (days)		5 [4, 8]	5 [4, 8]	5 [3, 9]	0.794 ^§^
RV4CSL %		−20.3 (4.4)	−22.5 (3.1)	−15.3 (2.3)	< 0.001^η^
RVFAC %		34.1 [26.2, 38.5]	36.0 (6.9)	25.6 (6.1)	< 0.001^η^
TAPSE mm	n (n missing)	45 (49) 23.7 [20.3, 25.5]	32 (35) 24.1 (2.9)	13 (14) 19.1 (4.6)	0.002^η^
S’ cm/s	n (n missing)	42 (52) 15.2 (3.4)	28 (39) 16.5 (2.7)	14 (13) 12.6 (3.3)	< 0.001^η^
RIMP	n (n missing)	38 (56) 0.42 [0.30, 0.54]	25 (42) 0.38 [0.3, 0.43]	13 (14) 0.62 [0.53, 0.87]	< 0.001^§^
LVEI Diastole	n (n missing)	51 (43) 1.04 [0.95, 1.54]	35 (32) 1.07 (0.23)	16 (11) 1.12 (0.27)	0.449^η^
LVEI Systole	n (n missing)	52 (42) 1.04 [0.94, 1.18]	36 (31) 1.06 (0.19)	16 (11) 1.09 (0.26)	0.653^η^
RV:LV Basal Diameter ED	n (n missing)	56 (38) 0.84 (0.13)	39 (28) 0.82 (0.12)	17 (10) 0.87 (0.13)	0.167^η^
Severe RV dilation (RV:LV > 1:1)	n (n missing)	90 (4) 23 (25.6%)	66 (1) 15 (22.7%)	24 (3) 8 (33.3%)	0.308^*^
Septal flattening	n (n missing)	90 (4) 9 (10%)	66 (1) 4 (6.1%)	24 (3) 5 (20.8%)	0.053^ω^
Severe ACP (severe RV dilation and septal flattening)	n (n missing)	89 (5) 7 (7.9%)	65 (2) 3 (4.6%)	24 (3) 4 (16.7%)	0.082^ω^
Subjective RV dysfunction^A^	n (n missing)	93 (1) 16 (17.2%)	67 (0) 7 (10.4%)	26 (1) 9 (34.6%)	0.012^ω^
Subjective LV dysfunction^A^	n (n missing)	92 (2) 11 (12%)	66 (1) 8 (12.1%)	26 (1) 3 (11.5%)	> 0.999^ω^

Data are presented as mean (SD), median [IQR] or n (%). Data are complete unless indicated by n (n missing)

A, Subjective RV and subjective LV dysfunction was visually assessed by the echocardiographer during imaging. Subjective RV or LV dysfunction was diagnosed by a reduction in thickening and motion of the RV myocardium or LV myocardium

*RVFWLS* Right Ventricular Free-wall longitudinal strain, *RV4CSL* Right Ventricular Four-Chamber longitudinal strain, *RVFAC* Right Ventricular Fractional Area Change, *TAPSE* Tricuspid Annular Plane Systolic Excursion, *S’* S’ wave velocity at the tricuspid annulus, *RIMP* Right ventricular Index of Myocardial Performance, *LVEI* Left Ventricular Eccentricity Index, *RV* Right Ventricle, *LV* Left Ventricle, *ED* End Diastole, *ACP* acute cor pulmonale

Between-group differences were assessed using Student’s *T* test (η), Mann–Whitney *U* test (§), Fisher’s Exact test (ω), and Pearson Chi-Square test (*)

### Patient characteristics

There was no difference in patient baseline demographics, co-morbidities, treatments before intubation, or acute comorbidities since hospital admission between normal and abnormal RVFWLS groups (Table 1). There was no significant difference in the prevalence of pulmonary thromboembolism (PTE) between the two groups (*p* = 0.097).

On the day of echocardiography, there was no difference in sequential organ failure assessment (SOFA) scores, acid–base-status, full blood count, C-reactive protein, or electrolytes between the normal and abnormal RVFWLS groups (*p* > 0.05 for all, Table 3 and Additional file 1: Table S2). Patients with abnormal RVFWLS had significantly higher hsTnI (*p* = 0.032), hsTnT (*p* < 0.001), and NT-proBNP (*p* = 0.004) compared to patients with normal RVFWLS. Patients with abnormal RVFWLS had higher heart rates (*p* = 0.028), were more often receiving vasopressors (*p* = 0.011) and had higher ventilatory driving pressures (*p* = 0.040).

**Table 3:** Patient characteristics on day of Echocardiography

		All (*n* = 94)	Normal RVFWLS (≤−20%) (*n* = 67)	Abnormal RVFWLS (> −20%) (*n* = 27)	*p* value
SOFA score	n (n missing)	93 (1) 8 [6, 10]	67 (0) 7 [6, 10]	26 (1) 9 [7, 10]	0.130^§^
Requirement for RRT on day of ECHO		12 (12.8%)	6 (9.0%)	6 (22.2%)	0.056^*^
*Cardiac biomarkers*					
hsTn I, ng/L	n (n missing)	57 (37) 13 [5, 39.5]	43 (24) 9 [4, 23]	14 (13) 39.5 [9, 146]	0.032^§^
hsTn T, ng/L	n (n missing)	35 (59) 18 [10, 29]	22 (45) 12.5 [9.3, 19.8]	13 (14) 27 [21.5, 47]	< 0.001^§^
Abnormal troponin^A^	n (n missing)	92 (2) 41 (44.6%)	65 (2) 21 (32.3%)	27 (0) 20 (74.1%)	< 0.001^*^
NT-proBNP, ng/L	n (n missing)	84 (10) 461 [109, 1798]	58 (9) 377 [165, 947]	26 (1) 1697 [302, 23271]	0.004^§^
Abnormal NT-proBNP^B^	n (n missing)	84 (10) 53 (63.1%)	58 (9) 33 (56.9%)	26 (1) 20 (76.9%)	0.079^*^
*Haemodynamic Parameters*					
HR, bpm	n (n missing)	92 (2) 79 [65, 96]	67 (0) 77 [63, 95]	25 (2) 84 [74, 99]	0.028^§^
Rhythm	n (n missing) Sinus AF/Flutter	92 (2) 88 (95.7%) 4 (4.3%)	67 (0) 64 (95.5%) 3 (4.5%)	25 (2) 24 (96%) 1 (4%)	0.703^ω^
Mean BP, mmHg	n (n missing)	89 (5) 77 [71, 87]	64 (3) 79 [72, 88]	25 (2) 76 [69, 86]	0.164^§^
CVP, mmHg	n (n missing)	59 (35) 7 [3, 12]	45 (22) 7 [2.5, 12]	14 (13) 8.5 [4.5, 12.5]	0.485^§^
*Drug Administration*					
Vasopressors		40 (42.6%)	23 (34.3%)	17 (63%)	0.011^*^
Inotropes		0 (0%)	0 (0%)	0 (0%)	NA
Anticoagulation					
Prophylactic		81 (86.2%)	61 (91%)	20 (74.1%)	0.097^*^
Therapeutic		11 (11.7%)	5 (7.5%)	6 (22.2%)	
None		2 (2.1%)	1 (1.5%)	1 (3.7%)	
Paralysis		47 (50%)	34 (50.7%)	13 (48.1%)	0.285^*^
*Ventilation*					
FiO_2_		0.55 [0.45, 0.7]	0.5 [0.45, 0.65]	0.55 [0.45, 0.8]	0.373^§^
Requirement for prone ventilation in previous 24 h		35 (37.2%)	27 (40.3%)	8 (29.6%)	0.484^*^
Plateau pressure, cmH_2_O	n (n missing)	48 (46) 25 (5.3)	34 (33) 24.6 (5.6)	14 (13) 26.1 (4.7)	0.397^η^
PAP, cmH_2_O	n (n missing)	91 (3) 26 [19, 30]	65 (2) 25 [19, 29]	26 (1) 27 [20, 31]	0.185^§^
Tidal volume, ml/kg (PBW)	n (n missing)	89 (5) 6.6 [5.9, 7.3]	64 (3) 6.5 [5.9, 7.2]	25 (2) 7.0 [5.9, 7.5]	0.335^§^
P/F ratio	n (n missing)	93 (1) 17.5 [12.9, 21.9]	67 (0) 17.5 [13.3, 21.8]	26 (1) 17.8 [12.3, 22,5]	0.918^§^
PEEP, cmH_2_O	n (n missing)	93 (1) 10 [8, 12]	66 (1) 10 [8, 12]	27 (0) 10 [6, 10]	0.110^§^
Respiratory rate (/minute)		25 [21, 28]	24.6 (5.0)	24.4 (5.7)	0.887^η^
Driving pressure, cmH_2_O	n (n missing)	48 (46) 13 [11 17.75]	34 (33) 12 [10, 16.25]	14 (13) 16.5 [12, 20]	0.040^§^
Dynamic compliance, ml/cmH_2_O	n (n missing)	48 (46) 28.1 [19.1, 39.7]	34 (33) 31.2 [21.6, 40.1]	14 (13) 21.2 [16.5, 35.1]	0.071^§^
Murray lung injury score	n (n missing)	82 (12) 2.8 [2.3, 3]	58 (9) 2.8 [2.2, 3]	24 (3) 2.8 [2.35, 3.2]	0.479^§^

Data are presented as mean (SD), median [IQR] or n (%). Data are complete unless indicated by n (n missing)

A, hsTnT ≥ 15 ng L^−1^ or hsTnI ≥ 34 ng L^−1^ for males; ≥ 16 ng L^−1^ for females. B, NT-proBNP  ≥ 300 ng L^−1^

*RVFWLS* Right Ventricular Free-wall longitudinal strain, *SOFA* Sequential organ failure Assessment, *RRT* Renal Replacement Therapy, *hsTn* High Sensitivity Troponin, *NT-proBNP* N-terminal pro B-type Natriuretic Peptide, *HR* Heart Rate, *AF* Atrial Fibrillation, *BP* Blood Pressure; *CVP* Central Venous Pressure, *FiO*_*2*_ Fraction of Inspired Oxygen, *PAP* Peak Airway Pressure, *PBW* Predicted Body Weight, *PEEP* Positive End Expiratory Pressure

Between-group differences were assessed using Student’s *T* test (η), Mann–Whitney *U* test (§), Fisher’s Exact test (ω), and Pearson Chi-Square test (*)

### Outcomes and survival analysis

**Fig. 1:**
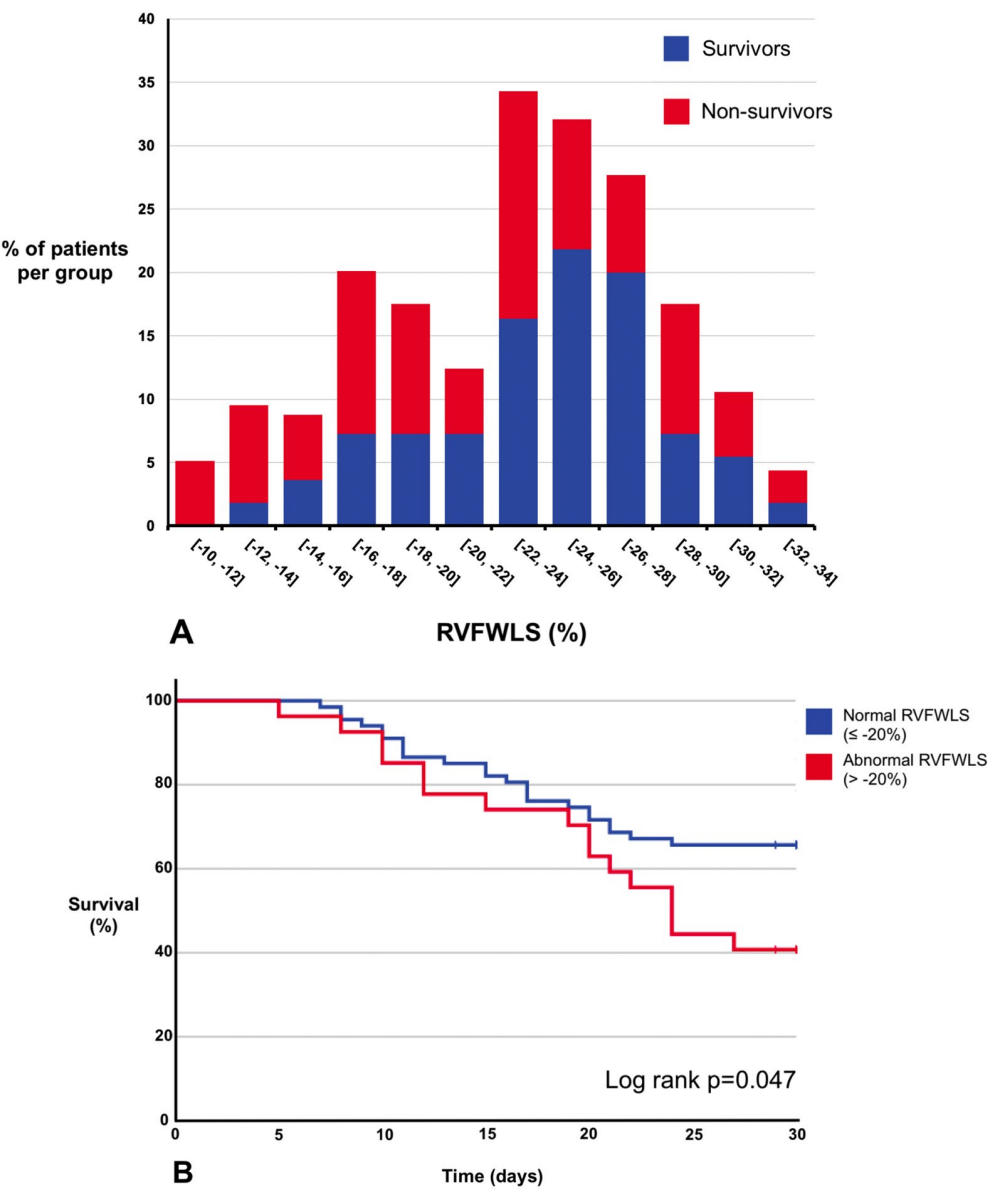
Survival Analysis. **A** Histogram displaying the distribution of Right Ventricular Free-Wall Longitudinal Strain (RVFWLS) in survivors and non-survivors. Percentage of patients in each 2% grouping of RVFWLS are shown by the histogram bars. Histogram bars from survivors and non-survivors are stacked upon each other. **B** Kaplan–Meier and log rank analysis of patients with normal RVFWLS (≤ −20%) (blue) compared to abnormal RVFWLS (> −20%) (red). Kaplan–Meier plot displays cumulative survival in the groups up to 30 days after ICU admission

At 30 days from ICU admission, 39 (41.5%) of all patients had died (Fig. 1A). Sixteen (59.3%) patients with abnormal RVFWLS died, compared to 23 (34.3%) patients with normal RVFWLS (*p* = 0.026). There was no difference between the two groups for the subsequent requirement for RRT, prone ventilation, or referral for ECMO (*p* > 0.280 for all, Table 4).

**Table 4:** Clinical outcomes at 30-day follow-up from ICU admission

	All (*n* = 94)	Normal RVFWLS (≤ −20%) (*n* = 67)	Abnormal RVFWLS (> −20%) (*n* = 27)	*p* value
Death	39 (41.5%)	23 (34.3%)	16 (59.3%)	0.026^*^
RRT	22 (23.4%)	15 (22.4%)	7 (25.9%)	0.760^*^
Prone ventilation	42 (44.7%)	32 (47.8%)	10 (37%)	0.578^*^
Referral for ECMO	12 (12.8%)	9 (13.4%)	3 (11.1%)	0.280^*^

Data are presented as n (%). Between-group differences were assessed using Pearson Chi-Square test (*)

*RVFWLS* Right Ventricular Free-wall Longitudinal Strain, *RRT* Renal replacement Therapy, *ECMO* Extracorporeal Membrane Oxygenation

Univariate analysis demonstrated that abnormal RVFWLS is associated with 30-day mortality (log-rank *p* = 0.047) (Fig. 1B). Multivariate cox regression demonstrated that abnormal RVFWLS is independently associated with 30-day mortality (Hazard Ratio 2.22 [1.14, 4.39], *p* = 0.020) (Table 5). Of the conventional RV echocardiography parameters, only abnormal RIMP was independently associated with mortality (*p* = 0.044, Additional file 1: Tables S4–S7).

**Table 5:** Multivariate Cox Regression predicting 30-day mortality including RVFWLS

Cox regression predicting 30-day mortality adjusting for remaining variables in table		
	HR (95% CI)	*p* value
Abnormal RVFWLS (> −20%)	2.22 (1.14, 4.39)	0.020
Age in years (per 1 year increase)	1.06 (1.01, 1.10)	0.009
Female Gender	0.86 (0.44, 1.71)	0.674
Non-white ethnicity	1.00 (0.28, 3.60)	0.994
APACHE II score on admission to ICU (per 1-score increase)	1.06 (1.00, 1.11)	0.040
Time from intubation to date of echo, in days (per 1 day increase)	0.91 (0.81, 1.03)	0.123

*N* = 89

*RVFWLS* Right Ventricular Free-wall Longitudinal Strain, *APACHE* Acute Physiology And Chronic Health Evaluation, *HR* Hazard Ratio

## Discussion

This is the largest prospective multicentre study investigating RV-STE in patients with COVID-19 requiring IMV and demonstrates the novel finding that abnormal RVFWLS is independently associated with 30-day mortality. Conversely, but in keeping with the findings of ARDS studies pre-COVID-19, conventional RV echocardiography parameters (RVFAC, TAPSE, and S’) were inconsistent in their association with mortality [22, 24].

Further supporting the utility of RVFWLS in ICU, the feasibility of RVFWLS was high (90.4%) even in this extremely challenging population. Echocardiography was performed predominantly by ICU clinicians in demanding circumstances; with poor quality echocardiography machines often assigned to the COVID-19 areas, the difficulty in obtaining acoustic windows in patients often undergoing ventilation with high airway pressures, combined with the hindrance of wearing cumbersome personal-protection-equipment. The high feasibility of RVFWLS despite these obstacles in the COVID-19 population would suggest that RVFWLS may be very feasible in other ICU populations. There was no difference in RVFWLS feasibility between images acquired by expert and non-expert echocardiographers, suggesting that acquiring images for RVFWLS is feasible in day-to-day ICU clinical practice. We would highlight that while we are suggesting acquisition of images of sufficient quality for RVFWLS analysis is highly feasible for ICU clinicians with a range of echocardiography experience, performing RV-STE analysis of these images is an advanced technique, and requires the reporter to have undergone dedicated training.

A recent large multicentre study in ICU patients with COVID-19 (of whom 69% were requiring IMV) identified a prevalence of RVD of 22.5% [25], and a meta-analysis of COVID-19 studies reported a prevalence of 20.4% [26], these reports are broadly similar to our findings, where prevalence of RVD was 28.7%.

The RVFWLS values in the current study are comparable to those reported by a pair of smaller studies investigating COVID-19 in patients requiring IMV. In the present study, the mean (SD) RVFWLS was −23.0% (5.2%), similar to −24.1% (6.9%) reported by Bleakley et al. [27]. Both our study and Bleakley et al. used TomTec software for RVFWLS analysis. In a similar clinical population, Gibson et al. reported a mean RVFWLS of -17% (6%) and a prevalence of RVD (RVFWLS  > −20%) of 65.6% [5]. Patients in this study did not appear to have more severe COVID-19 disease compared to ours; with similar SOFA scores, positive-end-expiratory-pressures/plateau pressures, and PaO_2_/FiO_2_ ratios. TAPSE and S’ were also similar in both studies. This more impaired RVFWLS and higher prevalence of RVD may partly be due to Gibson et al.’s inclusion of echocardiography imaging from 15 (46.9%) patients in the prone position (for which, as the authors highlight, RVFWLS has not been validated). In addition, the different strain software used (Epsilon) has been shown to report significantly less negative values (i.e., suggestive of poorer function) compared to the TomTec software that we used [28], possibly contributing to the disparity. This highlights a key challenge encountered with STE; different strain softwares, with unique proprietary algorithms, can generate different values. This issue has been addressed by a joint taskforce between industry and cardiovascular societies [18], with strain software slowly becoming more standardised [29]. For COVID-RV, we specifically chose to use vendor neutral TomTec strain software and were, therefore, able to perform analysis on images acquired from any manufacturer of ultrasound machine, allowing protocolised and reproducible central echo-lab analysis of images from across the country.

To differentiate between global cardiac (LV and RV) dysfunction and isolated RV dysfunction we collected data on the presence/absence of subjective LV dysfunction (shown to have good agreement with formal echocardiography LV assessment [30]). Given the low prevalence of subjective LV dysfunction (12%), and the finding of no difference between abnormal and normal RVFWLS groups, we believe that we have identified isolated RVD. This is in keeping with previous reports that have shown no difference in LV ejection fraction between abnormal and normal RVFWLS groups in patients with COVID-19 [4, 20].

We identified important exploratory associations between abnormal RVFWLS and putative mechanisms of RVD [5, 31–33]. We found association between RVD and myocardial injury, with higher troponin and NT-proBNP levels found in patients with abnormal RVFWLS. We also report significantly higher driving pressures in patients with abnormal RVFWLS (*p* = 0.040) with a trend toward lower lung compliances (*p* = 0.071), suggesting that injurious positive pressure ventilation may be a mechanism contributing to RVD. An association between high driving pressures and RVD has previously been identified in a non-COVID-19 ARDS population, supporting our results [6]. Perhaps unexpectedly, we did not find any association between abnormal RVFWLS and the incidence of PTE. During the early phase of the COVID-19 pandemic, the prevalence of PTE in ICU patients was reported as 16–31% [34, 35]. In contrast, we report a lower prevalence of radiologically confirmed/clinically suspected PTE of 8.5%. This may partly be due to the updated clinical guidance for the later phases of the pandemic; with more widespread use (and higher dosing) of pharmacological PTE prophylaxis, and the effects of immunotherapies, with studies showing a lower prevalence of PTE during later phases of the pandemic [36]. We note, however, that we did not systematically screen for PTE, and are, therefore, at risk of underreporting PTE prevalence.

Although we have identified important associations between possible causative mechanisms and abnormal RVFWLS, it is important to highlight that in general patient characteristics were similar between normal RVFWLS and abnormal RVFWLS groups. RVD is increasingly recognised as manifesting in a covert manner [37]. We have found that RVD can be subtle and difficult to diagnose clinically; however, it has a significant impact on survival. Clinical signs that may suggest a patient is at risk of RVD include high driving pressures, lower lung compliances, and vasopressor requirement. A high degree of clinical suspicion and actively seeking echocardiographic diagnosis is key to avoid missing subtle, but clinically important, RVD. There have been previous calls for systematic echocardiography screening of RVD in patients with ARDS [38], given our findings we would advocate this approach.

A recent meta-analysis of predominantly small retrospective COVID-19 studies concluded “RVD may represent one crucial marker for prognostic stratification in COVID‑19; [but] further prospective and larger [studies] are needed” [25]; our present study meets this need. Strengths of our study include its prospective design, that used images acquired predominantly by ICU clinicians with a range of echocardiography experience (reflecting day-to-day clinical practice). The comparative substantial limitations of retrospective design have been highlighted in a recent editorial on a multicentre echocardiography study in patients with COVID-19, with the authors commenting that “echocardiography exams were performed on clinical indication and not standardised which inferred some selection bias and some missing data” [39]. The echocardiography scans we obtained were study scans performed prospectively, giving an accurate representation of RVFWLS in COVID-19 patients undergoing IMV. The study recruited 24% of all patients with COVID-19 requiring IMV across ten Scottish ICUs during the study period meaning its results have broad applicability [40]. We adhered to a pre-published protocol and data analysis plan.

A limitation of our study is that we performed echocardiography at a single timepoint, and are at risk of underestimating the prevalence of RVD. Given that imaging occurred at different timepoints during different patients’ disease, we adjusted for time from intubation to echocardiography in multivariate analysis. We suggest that the fact echocardiography was obtained at different timepoints gives a broader representation of the effects that phase of disease has upon RV function. A second limitation is that we do not have information on chronic pulmonary arterial hypertension (PAH) or chronic RVD, which could represent a confounder (few patients in our study had previous echocardiography imaging or invasive measurement of pulmonary pressures). Our patient population included patients with chronic obstructive pulmonary disease (COPD), who may be at risk of chronic PAH/RVD; however, given that only 7.4% of patients had COPD (with equal distribution across normal and abnormal RVFWLS groups), we feel it is likely that any confounding effect would likely be small. A third limitation is that we did not measure LV-STE, a natural comparator for RV-STE. However, given that LV-STE requires a more advanced echocardiography image set, it was not feasible nor within the aims of this ICU clinician delivered study. In addition, given the focussed echocardiography image set, we were unable to include measures of pulmonary afterload, limiting our ability to elucidate the haemodynamic mechanisms underlying observed RVD. Finally, any associations identified between abnormal RVFWLS and possible causative mechanisms are at risk of type-1 error and should be viewed as exploratory only.

We report the novel finding that abnormal RVFWLS is independently associated with 30-day mortality in patients with COVID-19 requiring IMV. RVFWLS is highly feasible in this population, and can be analysed from images acquired by ICU clinicians who are both expert and non-expert echocardiographers, suggesting this technology may have utility in both COVID and non-COVID patients with severe respiratory failure requiring IMV. We have shown that RVD can manifest in a covert fashion in this cohort, a high degree of suspicion with systematic echocardiography to screen for RVD is advised. Preventive strategies, and early identification of RVD by RVFWLS analysis with prompt treatment, may be of therapeutic benefit in these patients.

## Supplementary Information

**Additional file 1: Figure S1.** Flow Diagram of Patient Recruitment. **Table S1**. RVFWLS Feasibility. **Table S2.** Laboratory Measurements on day of Echocardiography. **Figure S2.** Distribution of number of days from intubation to echocardiography. **Figure S3. **Incidence of 30-day mortality across RVFWLS Groups. **Figure S4.** Comparison of RVFWLS against TAPSE and S’. **Table S3.** Distribution of Conventional Right Ventricle Echocardiography Parameters Between Normal and Abnormal RVFWLS Groups. **Table S4.** Multivariate Cox Regression predicting 30-day mortality including Abnormal RVFAC. **Table S5.** Multivariate Cox Regression predicting 30-day mortality including Abnormal TAPSE. **Table S6.** Multivariate Cox Regression predicting 30-day mortality including Abnormal S’. **Table S7.** Multivariate Cox Regression predicting 30-day mortality including Abnormal RIMP.

## Data Availability

The data set used for this manuscript will be available from the corresponding author upon reasonable request.

## Abbreviations

AF: Atrial fibrillation
APACHE: Acute physiology and chronic health evaluation
APTT: Activated partial thromboplastin time
ARDS: Acute respiratory distress syndrome
A4C: Apical four-chamber
BE: Base excess
BP: Blood pressure
BSE: British Society of Echocardiography
COVID-19: Coronavirus disease 2019
COVID-RV: Right ventricular dysfunction in ventilated patients with COVID-19
CrCl: Creatinine clearance
CRP: C-reactive protein
CVP: Central venous pressure
ECMO: Extracorporeal membrane oxygenation
FAC: Fractional area change
FICE: Focused intensive care echocardiography
FiO2: Fraction of inspired oxygen
HR: Heart rate
hsTn: High sensitivity troponin
ICC: Intraclass correlation co-efficient
ICU: Intensive care unit
IMV: Invasive mechanical ventilation
LOA: Limits of agreement
LV: Left ventricular
LVEI: Left ventricular eccentricity index
NT-proBNP: N-terminal pro B-type natriuretic peptide
PAP: Peak airway pressure
PBW: Predicted body weight
PEEP: Peak end expiratory pressure
PT: Prothrombin time
PTE: Pulmonary thromboembolism
RIMP: Right ventricular index of myocardial performance
RRT: Renal replacement therapy
RV: Right ventricular
RVD: Right ventricular dysfunction
RVFWLS: Right ventricular free-wall longitudinal strain
RV4CSL: Right ventricular four-chamber longitudinal strain
S’: S’ Wave velocity at the tricuspid annulus
SD: Standard deviation
SOFA: Sequential organ failure assessment
STE: Speckle tracking echocardiography
TAPSE: Tricuspid annular plane systolic excursion
2D-CPA: 2D-Cardiac performance analysis
