# Supplementary material for: Right ventricular free wall longitudinal strain is independently associated with mortality in mechanically ventilated patients with COVID-19
Source: Ann Intensive Care. 2022 Nov 12;12:104. doi: 10.1186/s13613-022-01077-7 (PMC9652604; doi:10.1186/s13613-022-01077-7)
Supplement: Supplementary file 1 — Additional file 1: Figure S1. Flow Diagram of Patient Recruitment. Table S1. RVFWLS Feasibility. Table S2. Laboratory Measurements on day of Echocardiography. Figure S2. Distribution of number of days from intubation to echocardiography. Figure S3. Incidence of 30-day mortality across RVFWLS Groups. Figure S4. Comparison of RVFWLS against TAPSE and S’. Table S3. Distribution of Conventional Right Ventricle Echocardiography Parameters Between Normal and Abnormal RVFWLS Groups. Table S4. Multivariate Cox Regression predicting 30-day mortality including Abnormal RVFAC. Table S5. Multivariate Cox Regression predicting 30-day mortality including Abnormal TAPSE. Table S6. Multivariate Cox Regression predicting 30-day mortality including Abnormal S’. Table S7. Multivariate Cox Regression predicting 30-day mortality including Abnormal RIMP. [file 13613_2022_1077_MOESM1_ESM.docx]

Title: Right Ventricular Free Wall Longitudinal Strain is Independently Associated with Mortality in Mechanically Ventilated Patients with COVID-19

Journal: Annals of Intensive Care

Dr James McErlane^1,2^, Dr Philip McCall^1,2^, Dr Jennifer Willder^3^, Professor Colin Berry^4^, Dr Ben Shelley^1,2^ on behalf of the COVID-RV investigators.

1. Anaesthesia, Critical Care & Peri-operative Medicine Research Group, University of Glasgow, UK.
2. Department of Anaesthesia, Golden Jubilee National Hospital, Clydebank, UK.
3. West of Scotland School of Anaesthesia, NHS Education for Scotland, Glasgow, UK.
4. Institute of Cardiovascular and Medical Sciences, University of Glasgow, UK.

Corresponding author: Dr James McErlane

[James.mcerlane@glasgow.ac.uk](mailto:James.mcerlane@glasgow.ac.uk)

ORCID ID: 0000-0002-0490-8425

Supplementary Figure 1. Flow Diagram of Patient Recruitment

Supplementary Table 1 RVFWLS Feasibility

| RVFWLS Feasibility | | |
| --- | --- | --- |
| Group | Number of echo scans of sufficient quality for RVFWLS/  Total number of echo scans performed (%) | p-value |
| All | 94/104 (90.4%) |  |
| *Echo speciality* | | |
| Critical care | 72/80 (90%) | >0.999^ω^ |
| Cardiac physiologist/departmental echocardiographer | 22/24 (91.7%) |  |
| *Echo accreditation* | | |
| None | 13/14 (92.9%) | 0.672* |
| FICE | 1/1 (100%) |  |
| FICE mentor | 33/35 (94.3%) |  |
| BSE Critical Care accreditation | 4/4 (100%) |  |
| BSE full accreditation | 43/50 (86.0%) |  |

Data are presented as n (%). Between-group differences were assessed using Fisher’s Exact test (ω) and Pearson Chi-Square test (*). Echo = echocardiography; RVFWLS= Right Ventricular Free-Wall Longitudinal Strain; FICE = Focused Intensive Care Echocardiography; BSE = British Society of Echocardiography.

Supplementary Table 2 Laboratory Measurements on day of Echocardiography

| Laboratory Measurements on day of Echocardiography | | | | | |
| --- | --- | --- | --- | --- | --- |
|  | | All (n=94) | Normal RVFWLS (≤-20%) (n=67) | Abnormal RVFWLS  (>-20%) (n=27) | p-value |
| [H+], nmol/L | n (n missing) | 88 (6)  39.7 [36, 46.9] | 65 (2)  40 [37, 46.9] | 23 (4)  39 [34, 48] | 0.700^§^ |
| PaO_2_, kPa | n (n missing) | 93 (1)  9.2 [8.5, 10.1] | 67 (0)  9.1 [8.5, 10] | 26 (1)  9.6 [8.6, 10.6] | 0.251^§^ |
| PaCO_2_, kPa | n (n missing) | 89 (5)  7 [6.1, 8.0] | 65 (2)  7 [6.2, 8] | 24 (3)  6.5 [5.9, 8.6] | 0.781^§^ |
| BE, mmol | n (n missing) | 90 (4)  6.1 (6.6) | 66 (1)  6.4 (6.2) | 24 (3)  5.1 (7.7) | 0.404^η^ |
| Bicarbonate, mmol/L | n (n missing) | 90 (4)  31.7 [27, 41.9] | 65 (2)  31.9 [28, 35.6] | 25 (2)  30 [25, 36.5] | 0.353^§^ |
| Haemoglobin, g/dL | | 10.9 [9.6, 12.1] | 11.1 [10.1, 12.2] | 10.3 [8.9, 12.9] | 0.148^§^ |
| Neutrophils, x10^9^/L | | 10.4 [8.5, 14.2] | 10.2 [8.4, 13.1] | 12.3 [8.6, 19.4] | 0.058^§^ |
| Lymphocytes, x10^9^/L | | 1 [0.6, 1.4] | 1 [0.5, 1.3] | 1.1 [0.7, 1.6] | 0.200^§^ |
| Platelets, x10^9^/L | | 272 [211, 342] | 274 [213, 338] | 270 [207, 373] | 0.809^§^ |
| CRP, mg/L | | 52.5 [9, 158] | 54 [9, 169] | 51 [12, 119] | 0.877^§^ |
| D-Dimer, mg/L FEU | n (n missing) | 66 (28)  1432.5 [652-2811] | 46 (21)  1224 [534-3079] | 20 (7)  1918.5 [952-2682] | 0.230^§^ |
| PT, seconds | | 11.7 [11, 13] | 11.6 [11, 13] | 11.8 [11, 13.3] | 0.696^§^ |
| APTT, seconds | n (n missing) | 93 (1)  26 [24-30] | 66 (1)  26 [23.3-29] | 27 (0)  28.3 [25, 33] | 0.099^§^ |
| Creatinine, μmol/L | | 67.5 [52.8, 105] | 65 [39.8, 97] | 88 [57, 108] | 0.176^§^ |
| CrCl of patients not receiving RRT (ml/min) | n (n missing) | 79 (15)  138.9 [84.1, 176.4] | 60 (7)  140 [87.9, 174.4] | 19 (8)  133 [74.9, 176.8] | 0.383^§^ |

Data are presented as mean (SD), median [IQR] or n (%). Data are complete unless indicated by n (n missing).

Between-group differences were assessed using Student’s T-test (η), Mann-Whitney U test (§)

RVFWLS = Right Ventricular Free-wall longitudinal strain; BE = Base Excess; CRP = C-Reactive Protein; PT = Prothrombin Time; APTT = Activated Partial Thromboplastin Time; CrCl = Creatinine Clearance


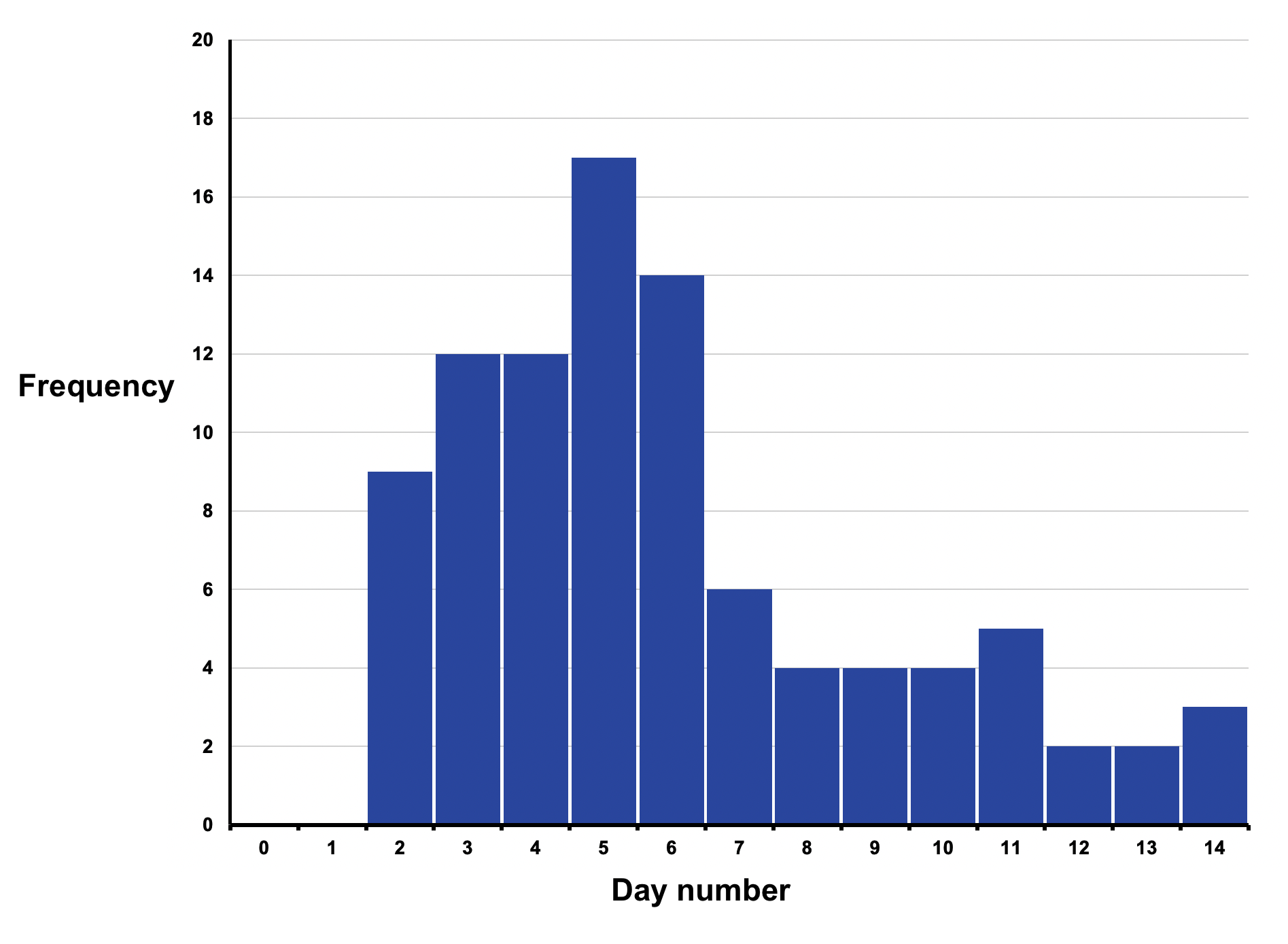
 Supplementary Figure 2. Distribution of number of days from intubation to echocardiography

Histogram displaying the distribution in the time variability from intubation to echocardiography. Intubation occurred on day 0, echocardiography occurred at earliest on day 2 (>48h after intubation).


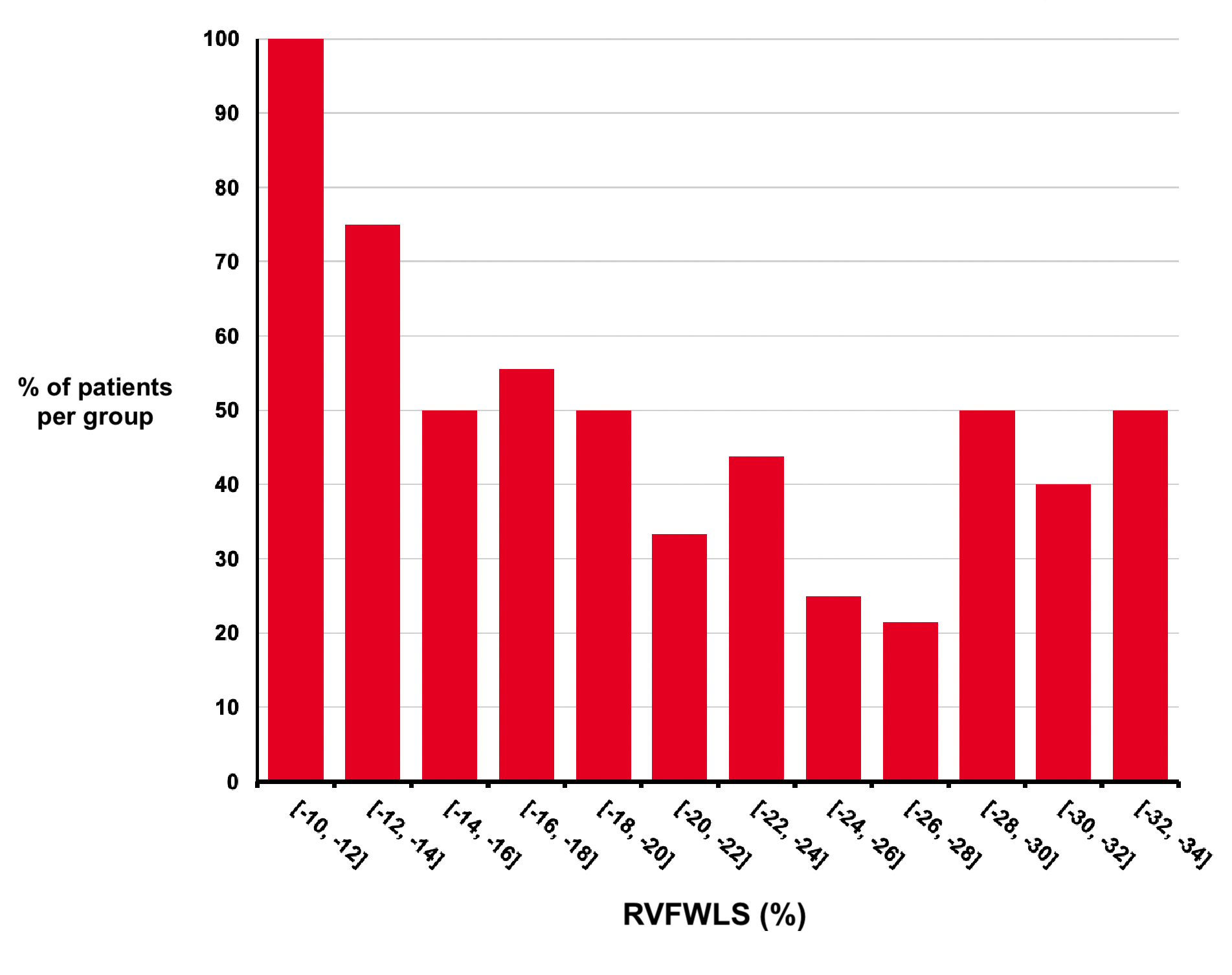


Supplementary Figure 3. Incidence of 30-day mortality across RVFWLS Groups

Histogram displaying the incidence of 30-day mortality within the 2% grouping of Right Ventricular Free-Wall Longitudinal Strain (RVFWLS).


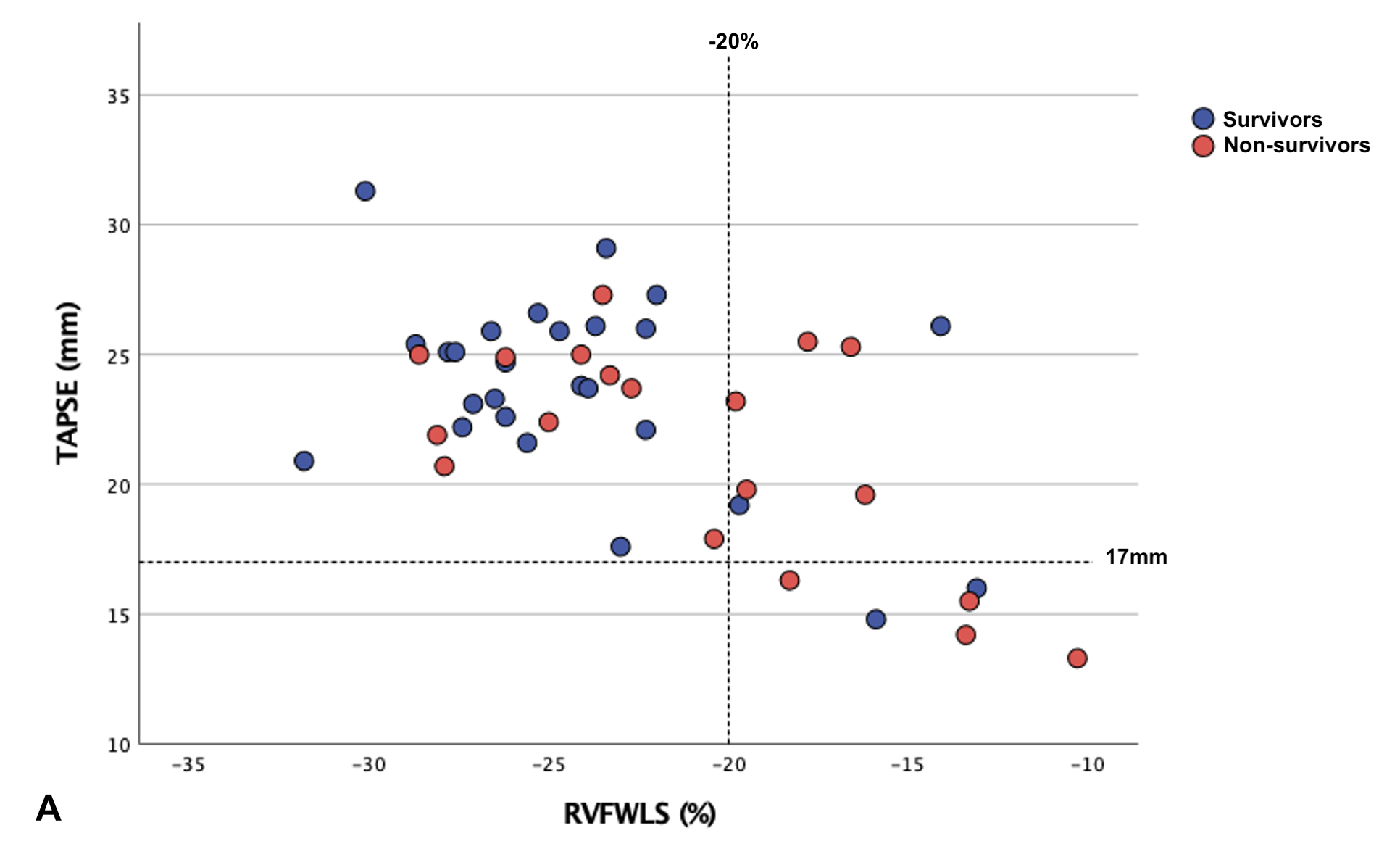


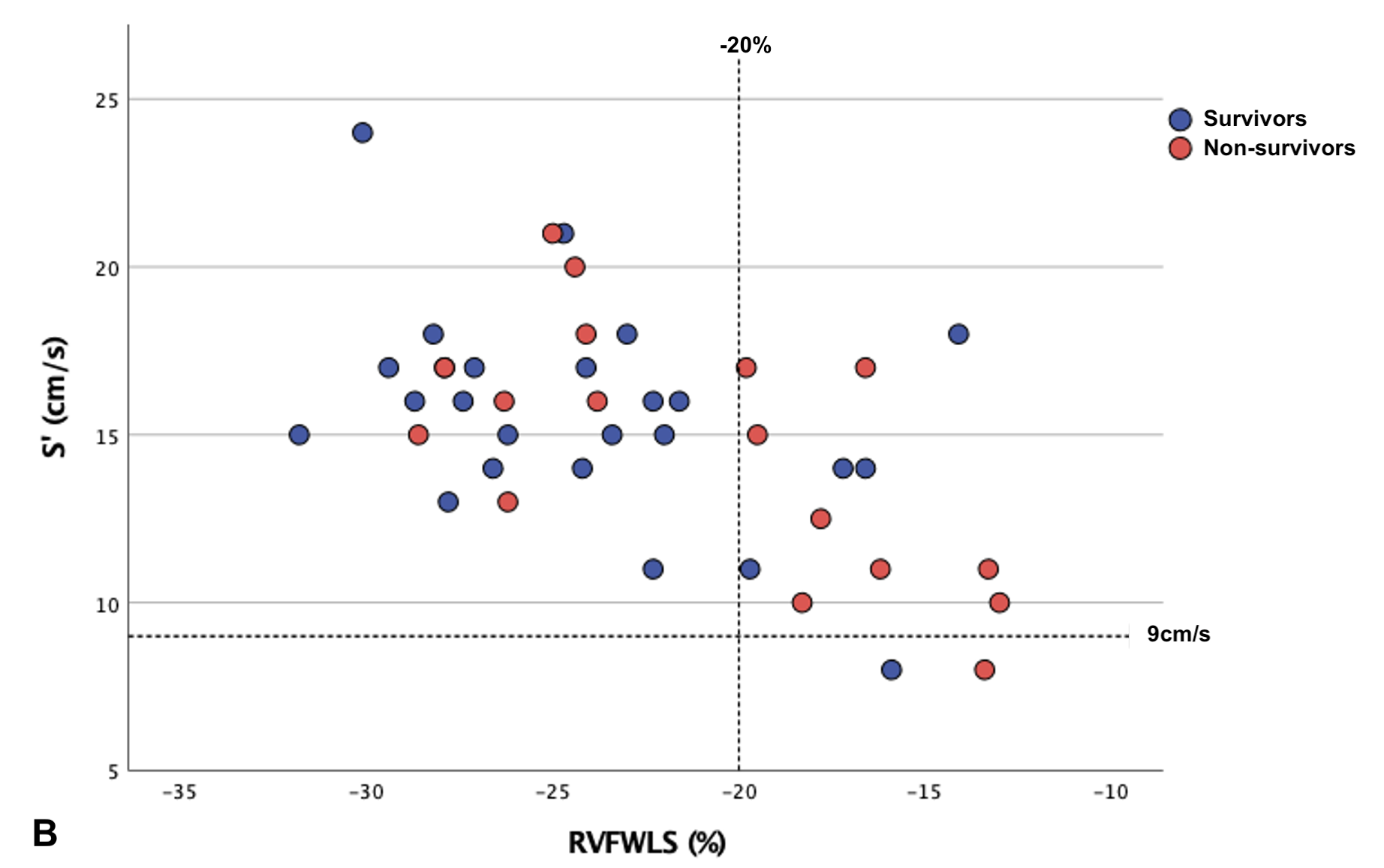


Supplementary Figure 4. Comparison of RVFWLS against TAPSE and S’

Scatter plots comparing Right Ventricular Free-Wall Longitudinal Strain (RVFWLS) against conventional RV parameters (Tricuspid annular plane systolic excursion **A** [n=45], S’ wave velocity at the tricuspid annulus **B** [n=42]). Survivors at 30-days post-ICU admission are shown in red, non-survivors in blue. Abnormal cuts off^1^ are shown as a dashed line.

Supplementary Table 3 Distribution of Conventional Right Ventricle Echocardiography Parameters Between Normal and Abnormal RVFWLS Groups

| Echo parameter | Abnormal cut-off^[[1]](#footnote-1)^ | Total | Normal RVFWLS (≤-20%) | Abnormal RVFWLS (>-20%) |
| --- | --- | --- | --- | --- |
| Abnormal RVFAC | Males <30%, Females <35% | 36/94 | 13/67 (19.4%) | 23/27 (85.2%) |
| Abnormal TAPSE | <17mm | 6/45 | 0/32 (0%) | 6/13 (46.2%) |
| Abnormal S’ | <9 cm/second | 2/42 | 0/28 (0%) | 2/14 (14.3%) |
| Abnormal RIMP | >0.54 | 9/38 | 0/25 (0%) | 9/13 (69.2%) |

RVFWLS = Right Ventricular Free-wall longitudinal strain; RVFAC = Right Ventricular Fractional Area Change; TAPSE = Tricuspid Annular Plane Systolic Excursion, S’ = S’ wave velocity at the tricuspid annulus; RIMP = Right ventricular Index of Myocardial Performance

Supplementary Table 4 Multivariate Cox Regression predicting 30-day mortality including Abnormal RVFAC

| Table 2. Cox regression predicting 30-day mortality adjusting for remaining variables in table | | |
| --- | --- | --- |
|  | HR (95% CI) | p-value |
| Abnormal RVFAC (<30% males, <35% females)^1^ | 1.31 (0.66, 2.59) | 0.445 |
| Age in years (per 1-year increase) | 1.05 (1.01, 1.10) | 0.015 |
| APACHE II score on admission to ICU (per 1-score increase) | 1.07 (1.01, 1.13) | 0.023 |

RVFAC = Right Ventricular Fractional Area Change; APACHE = Acute Physiology And Chronic Health Evaluation; HR = Hazard Ratio. N=89

Supplementary Table 5 Multivariate Cox Regression predicting 30-day mortality including Abnormal TAPSE

| Table 3. Cox regression predicting 30-day mortality adjusting for remaining variables in table | | |
| --- | --- | --- |
|  | HR (95% CI) | p-value |
| Abnormal TAPSE (<17mm)^1^ | 1.73 (0.56, 5.32) | 0.339 |
| Age in years (per 1-year increase) | 1.05 (0.98, 1.13) | 0.155 |
| APACHE II score on admission to ICU (per 1-score increase) | 1.04 (0.96, 1.12) | 0.400 |

TAPSE = Tricuspid Annular Plane Systolic Excursion; APACHE = Acute Physiology And Chronic Health Evaluation; HR = Hazard Ratio. N=44

Supplementary Table 6 Multivariate Cox Regression predicting 30-day mortality including Abnormal S’

| Table 4. Cox regression predicting 30-day mortality adjusting for remaining variables in table | | |
| --- | --- | --- |
|  | HR (95% CI) | p-value |
| Abnormal S’ (<9 cm/second)^1^ | 1.21 (0.16, 9.41) | 0.853 |
| Age in years (per 1-year increase) | 1.06 (0.99, 1.13) | 0.126 |
| APACHE II score on admission to ICU (per 1-score increase) | 1.05 (0.96, 1.15) | 0.326 |

S’ wave velocity at the tricuspid annulus; APACHE = Acute Physiology And Chronic Health Evaluation; HR = Hazard Ratio. N=39

Supplementary Table 7 Multivariate Cox Regression predicting 30-day mortality including Abnormal RIMP

| Table 5. Cox regression predicting 30-day mortality adjusting for remaining variables in table | | |
| --- | --- | --- |
|  | HR (95% CI) | p-value |
| Abnormal RIMP (>0.54)^1^ | 3.19 (1.03, 9.85) | 0.044 |
| Age in years (per 1-year increase) | 1.07 (0.99, 1.16) | 0.082 |
| APACHE II score on admission to ICU (per 1-score increase) | 1.03 (0.92, 1.16) | 0.580 |

RIMP = Right ventricular Index of Myocardial Performance; APACHE = Acute Physiology And Chronic Health Evaluation; HR = Hazard Ratio. N=36

1. Zaidi A, Knight DS, Augustine DX, Harkness A, Oxborough D, et al. Education Committee of the British Society of Echocardiography. Echocardiographic assessment of the right heart in adults: a practical guideline from the British Society of Echocardiography. Echo Res Pract. 2020 Feb 27;7(1):G19-G41. <https://doi.org/10.1530/ERP-19-0051> [↑](#footnote-ref-1)
